# The Impact of Metal Ion Exposure on the Cellular Behavior of Human Osteoblasts and PBMCs: In Vitro Analyses of Osteolytic Processes

**DOI:** 10.3390/ma10070734

**Published:** 2017-07-03

**Authors:** Anika Jonitz-Heincke, Jenny Tillmann, Annett Klinder, Simone Krueger, Jan Philippe Kretzer, Paul Johan Høl, Alexander C. Paulus, Rainer Bader

**Affiliations:** 1 Biomechanics and Implant Technology Research Laboratory, Department of Orthopaedics, University Medical Center Rostock, 18057 Rostock, Germany; jenny.tillmann@gmx.net (J.T.); annett.klinder@med.uni-rostock.de (A.K.); simone.krueger@med.uni-rostock.de (S.K.); rainer.bader@med.uni-rostock.de (R.B.); 2 Clinic for Orthopaedics and Trauma Surgery, Heidelberg University Hospital, 69118 Heidelberg, Germany; philippe.kretzer@med.uni-heidelberg.de; 3 Department of Orthopaedic Clinic, Biomatlab, Haukeland University Hospital, 5021 Bergen, Norway; paul.johan.hol@helse-bergen.no; 4 Department of Clinical Medicine, Biomaterials, University of Bergen, 5021 Bergen, Norway; paul.hol@uib.no; 5 Department of Orthopaedic Surgery, University Hospital Munich (Campus Grosshadern), 81377 Munich, Germany; alexander.paulus@med.uni-muenchen.de

**Keywords:** osteolysis, corrosion, inflammation, osteoblasts, PBMCs, metal ions, CoCr, cytokines

## Abstract

Osteolysis in the periprosthetic tissue can be caused by metallic wear particles and ions that can originate from implant surface corrosion. These products influence cellular behavior and stimulate the expression of proinflammatory cytokines. The purpose of this study was to evaluate the impact of CoCr29Mo6 ions on cell survival, differentiation, and cytokine expression in human osteoblasts and peripheral blood mononuclear cells (PBMCs). Thus, we exposed cells with a mixture of 200 µg/L ion solution and determined cell viability and apoptosis/necrosis. Gene expression analyses of osteoblastic and osteoclastic differentiation markers as well as pro-osteolytic mediators (*IL-6*, *IL-8*, *TNF-α*, *MCP-1*, *MMP1*, *TIMP1*) were performed. These markers were also investigated in mixed cultures of adherent and non-adherent PBMCs as well as in co-cultures of human osteoblasts and PBMCs. The ion solution induced necrosis in osteoblasts and PBMCs in single cultures. All examined mediators were highly expressed in the co-culture of osteoblasts and PBMCs whereas in the single cell cultures only *IL-6*, *IL-8*, and *MMP1* were found to be stimulated. While the applied concentration of the CoCr29Mo6 ion solutions had only marginal effects on human osteoblasts and PBMCs alone, the co-culture may provide a comprehensive model to study osteolytic processes in response to Co and Cr ions.

## 1. Introduction

Total joint replacement is one of the most performed operations in orthopaedic surgery whereby degenerative and painful articular surfaces are substituted by artificial anatomically-shaped endoprostheses [1]. Tens of thousands of hip and knee replacements are carried out each year in Europe and the United States improving the patient’s quality of life [1]. Due to the ageing population in the industrial countries and the increasing number of endoprosthetic implants, the wear-related pathology has gained more importance. Extensive research and collaborative work of orthopaedic surgeons has led to the development of innovative bearing surfaces, however, no single material is yet without fault. So far, it is not avoidable for articulating surfaces to generate wear debris from the endoprosthesis. Periprosthetic osteolysis, as a result of the wear products, is thought to be the main cause for implant failure, resulting in a reduced life time of the endoprostheses [1]. The presence of wear particles in the periprosthetic tissue is caused by abrasion, corrosion, or a synergistic combination of both [1]. By analysing the effects of abrasive wear particles which were artificially generated in the interface between bone cement and hip stems, an upregulation of osteolytic processes in human osteoblasts and macrophages was observed in vitro [2,3,4,5].

Most metals and metal alloys used for orthopaedic implants are protected against electrochemical corrosion by a passifying oxide layer. Nonetheless, this oxide layer can be interrupted by mechanical stress and electrochemical or cellular reactions, resulting in corrosion processes within the physiological environment by the release of wear particles and metal ions [6,7]. Some patients with aseptic implant loosening showed elevated amounts of metal ion concentrations within the surrounding tissue, in other organs, as well as body fluids, suggesting a direct influence of corrosion and local biochemical processes [6,7]. Nevertheless, the release of metal ions might not directly initiate osteolysis and aseptic loosening; in fact, these ions must react with biomolecules to become more bioactive and destructive [8]. 

The main metallic alloy for articulating implant components is cobalt-chromium-molybdenum (CoCrMo). Although the ionic forms of Co and Cr are essential elements for biological functions, these microelements can cause local to systemic toxicity reactions at high concentrations [9]. The toxicity of Co and Cr also depends on their oxidative state, i.e., Cr(III) and Co(II) have been found in biological fluids of metal-on-metal (MoM) bearing patients [9]. An ion concentration of 5–100 µg/L of Cr and 5–300 µg/L of Co in the plasma and tissue from patients with MoM bearings undergoing revision surgery was reported [9]. The presence of CoCr corrosion leads to increased inflammation in the local tissue [10] associated with tissue necrosis as well as macrophage and lymphocytic infiltration [9]. Besides hypersensitivity reactions and oxidative stress, these metal ions are known to stimulate the secretion of pro-inflammatory cytokines resulting in osteolysis [8]. Several in vitro studies reported the relation between interleukin (IL-)1β and tumour necrosis factor (TNF-)α release by macrophages in response to Co/Cr ion exposure in a dose- and time-dependent manner [11,12,13]. Additionally, toxic effects of metal ions from CoCr alloys against osteoblasts were shown whereas Co and Cr were less toxic than vanadium, nickel, or iron [8]. 

The main purpose of our present in vitro study was to evaluate the effect of metal ions on bone formation and degradation as well as the release of pro-inflammatory mediators by human osteoblasts and peripheral blood mononuclear cells (PBMCs). Hence, we generated an ion solution from a CoCr29Mo6 alloy and used a total ion concentration of 200 µg/L in our in vitro experiments. The concentration used in the experiment here is based on examinations of joint aspirates of patients undergoing revision surgery that show average concentrations of 200–250 µg/L in the joint fluid [14]. 

When using only one cell type for studying the effects of the wear products in vitro, these culture models are very static and do not reflect the systemic and chronic exposure of the dynamic response in vivo [15]. While wear products do not only induce a local response but also a systemic one [16], more complex cell culture models have to be established to understand the different interactions of cells. Up to now, there are only a few published studies dealing with co-culture cell models for analysing osteolytic processes in response to wear particles [17,18,19,20,21]. Therefore, we intended to establish the co-culture of human osteoblasts and PBMCs to analyse the impact of cellular interactions in response to metal ion exposure compared to single cell culture experiments.

## 2. Results

### 2.1. Effects of CoCr29Mo6 Ions in Single Cultures of Human Osteoblasts and PBMCs

#### 2.1.1. Viability

**Figure 1:**
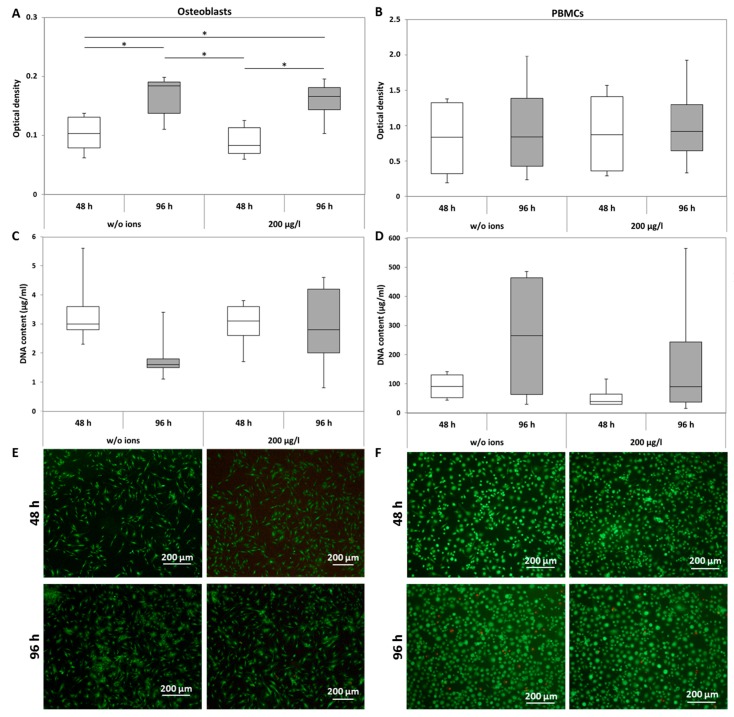
Analysis of cell viability of human osteoblasts and peripheral blood mononuclear cells (PBMCs) after exposure with 200 µg/L CoCr29Mo6 ion solution. Cells were transferred into standard culture plates allowing adherence over 24 h (osteoblasts) or 72 h (PBMCs). Afterwards, the cell culture medium was replaced by the ion solution. After 48 h and 96 h, metabolic activity was determined by the water soluble tetrazolium salt (WST-1) assay (**A**,**B**); Cell proliferation was quantified by DNA measurements (**C**,**D**) and live-dead stainings were carried out (**E**,**F**). Data are shown as box plots (*n* = 4). Significances between groups were calculated with the Mann-Whitney-U-Test (* *p* ≤ 0.05, treated vs. untreated).

The metabolic activity of human osteoblasts was not affected by CoCr29Mo6 ions at a concentration of 200 µg/L compared to the unstimulated cells. Comparing both time points, significantly elevated values were determined for both untreated (*p* = 0.03) and treated (*p* = 0.017) cells (Figure 1A). In human PBMC cultures, no significant differences between treated and untreated as well as between time points were detected (Figure 1B). The quantification of DNA contents revealed no significant differences between treated and untreated cells for both osteoblasts and adherent PBMCs (Figure 1C,D), suggesting that increased metabolic activity is due to differentiation rather than proliferation in human osteoblasts. In order to verify this assumption at least qualitatively, live-dead staining was carried out (Figure 1E (osteoblasts) and Figure 1F (PBMCs)). While the number of stained cells was not counted, qualitative observations showed no differences in the vital cell number between ion exposure and unstimulated cells. Only a few dead cells were detected. For PBMCs, an increase of adherent, sprawling cells in treated and untreated cultures was visible over time, indicating the differentiation potential of the monocyte fraction of the PBMCs.

#### 2.1.2. Apoptosis and Necrosis

The influence of CoCr29Mo6 ions on apoptosis and necrosis was determined in human osteoblasts and adherent PBMCs. The assay procedure is based on the determination of cytoplasmic histone-associated DNA fragments after induced cell death. According to the user’s manual, necrosis was quantified in the supernatant of the cell cultures whereas apoptosis was determined in the cell lysates of adherent cells. 

**Figure 2:**
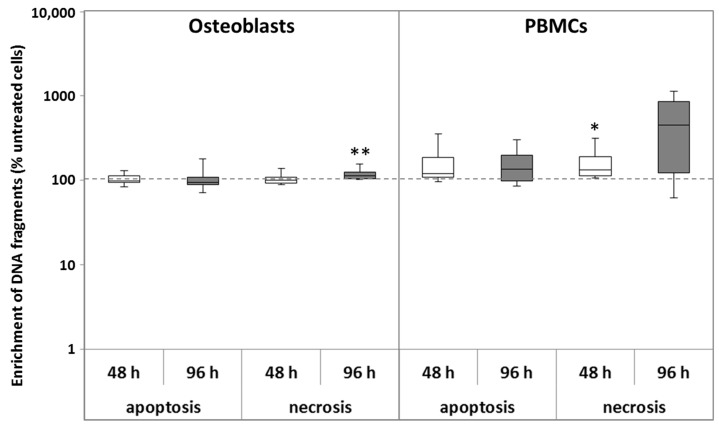
Analysis of apoptosis and necrosis of human osteoblasts and PBMCs after exposure with 200 µg/L CoCr29Mo6 ion solution. Cells were transferred into standard culture plates allowing adherence over 24 h (osteoblasts) or 72 h (PBMCs). Afterwards, the cell culture medium was replaced by the ion solution. After 48 h and 96 h, necrosis was determined within cell culture supernatants. For apoptosis, cells were lysed for 30 min at room temperature. Absorbance was measured at 405 nm (reference wave length: 490 nm) and the apoptosis and necrosis rates were then normalised to the untreated control at each time point. Data are shown as box plots (*n* = 4). Significances between groups were calculated with the Mann-Whitney-U-Test (* *p* ≤ 0.05, ** *p* < 0.01, treated vs. untreated).

The exposure to CoCr29Mo6 ions did not induce apoptosis in human osteoblasts. In contrast, a slight increase of apoptotic cells could be detected in PBMC cultures. An increase of necrotic cells was visible for both cell types. For osteoblasts, this increase was detected after 96 h (*p* = 0.008) whereas in PBMCs, necrosis was induced earlier (48 h; *p* = 0.029) and was much more enhanced after 96 h. Due to high inter-individual variations, the observed upregulation of necrosis in PBMCs did not reach significance after 96 h (Figure 2). 

#### 2.1.3. Osteoblastic Differentiation and Induction of Osteoclastic Differentiation Markers

Ions from CoCr29Mo6 significantly induced the expression of collagen 1 mRNA after 96 h of incubation compared to the unstimulated cells (*p* = 0.016). This increase was paralleled by elevated levels for the collagen 1 protein after 96 h exposure compared to the unstimulated cells (Table 1). No significant changes in alkaline phosphatase (*ALP*) mRNA, osteocalcin (*OC*) mRNA, and osteoprotegerin (*OPG*) mRNA expression rates were detected in ion-stimulated osteoblastic cells. The increase of receptor activator of nuclear κ B ligand (*RANKL*) mRNA after 48 h compared to unstimulated cells declined. 

**Figure 3:**
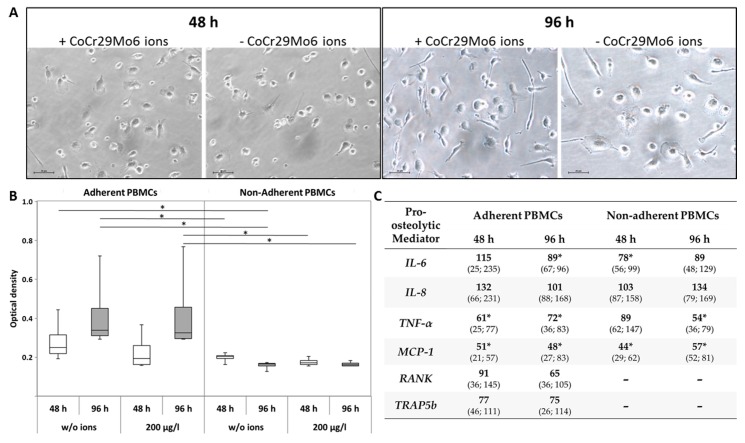
Influence of CoCr29Mo6 ions in the mixed culture of adherent and non-adherent PBMCs. (**A**) Light microscopy of PBMCs (bar: 20 µm); (**B**) Metabolic activity of PBMCs; (**C**) Gene expression analysis of pro-osteolytic mediators in cultures of adherent/non-adherent PBMCs. Cells were transferred in standard cell culture plates exposing them with 200 µg/L CoCr29Mo6 ions. After 48 h and 96 h, analyses of cell culture experiments were done; (**B**) Data are shown as box plots (*n* = 4). Significances between groups were calculated with the Mann-Whitney-U-Test (* *p* ≤ 0.05, adherent vs. non-adherent PBMCs); (**C**) Data are shown as median and minimum/maximum values (*n* = 4; all in %). Significances between groups were calculated with the Mann-Whitney-U-Test (* *p* ≤ 0.05, treated vs. untreated).

In PBMC cultures, significantly reduced expression rates of *RANK* mRNA were shown after treatment with CoCr29Mo6 ions (*p* = 0.029 (48 h); *p* = 0.029 (96 h); *p* = 0.029 (48 h vs. 96 h)) (Figure 3B). The mRNA expression rate of the osteoclastic differentiation marker tartrate resistant acid phosphatase *(TRAP)5b* was also significantly reduced in PBMCs after exposure to the ions (both: *p* = 0.029) (Table 1).

#### 2.1.4. Gene Expression of Pro-Osteolytic Mediators

Observed changes in matrix metalloprotease *(MMP)1* synthesis rates in human osteoblasts and PBMCs did not reach significance when compared to the untreated cells. Tissue inhibitor of MMP *(TIMP)1*—the natural inhibitor of *MMP1*—was initially enhanced (*p* = 0.029) in osteoblasts whereas in human PBMCs mRNA levels were not influenced by CoCr29Mo6 ions (Table 2). 

*IL-6* and *IL-8* mRNA levels in human osteoblasts were induced after 48 h of ion exposure (*IL-6*: *p* = 0.008) and *IL-8* mRNA was decreased after 96 h (*p* = 0.032). Values for *IL-6* after 96 h were similar compared to mRNA expression levels in the control cells. *IL-8* mRNA levels were also upregulated in human PBMCs at both time points (*p* = 0.029 (48 h)), however, there were no significant changes in *IL-6* mRNA expression (Table 2). Changes in *TNF-α* mRNA expression were not significant. 

Monocyte chemoattractant protein *(MCP-)1* gene expression rate was unaffected in osteoblasts. In PBMCs, an initial downregulation (*p* = 0.029) of *MCP-1* mRNA was detected in comparison to unstimulated PBMCs. After 96 h, an upregulation of *MCP-1* was observed which did not reach significance due to high inter-individual variations (Table 2).

### 2.2. Influence of CoCr29Mo6 Ions in Co-Cultures

#### 2.2.1. Adherent and Non-Adherent PBMCs

Since it was reported that macrophages and lymphocytes are directly involved in wear-mediated inflammatory reactions [9], we investigated the effect of CoCr29Mo6 ions on cell adhesion of the monocytic fraction in a mixed culture of isolated PBMCs which contained both, monocytes as well as lymphocytes. Additionally, effects on cell viability and on the gene expression of pro-osteolytic mediators in adherent and non-adherent cells were analysed. After 48 h of exposure, light microscopy revealed little differences in cell attachment of monocytes, whereas after 96 h, more sprawling PBMCs were visible in the ion-treated cultures (Figure 3A). We also determined an upregulation of metabolic activity in treated and untreated adherent cells over time in the water soluble tetrazolium salt (WST-1) assay. Significantly higher values were measured for adherent cells compared to cells in suspension (all: *p* = 0.029) at each time point and for each stimulation group (Figure 3B). 

Results for the analysis of mRNA expression levels for pro-osteolytic mediators are shown in Figure 3C. *IL-6* gene expression rates were similar to those detected in PBMCs in single cell culture after ion exposure at both time points. The cells in suspension showed a downregulation of *IL-6* mRNA (*p* = 0.029 (48 h)). *IL-8* gene expression was not significantly affected. *TNF-α* and *MCP-1* mRNA levels were clearly reduced in both cell types. For the latter, significant differences in mRNA levels of *MCP-1* were detected in adherent and suspension cells at both time points (all: *p* = 0.029). In adherent PBMCs, significant differences in *TNF-α* gene expression were detectable at both time points (both: *p* = 0.029) whereas a significant downregulation in non-adherent PBMCs could be noticed after 96 h (*p* = 0.029). 

Compared to the single cell culture in which a significant reduction of mRNA levels of *RANK* and *TRAP5b* were observed in PBMCs cultures after ion treatment, an upregulation in the mixed culture was clearly notable (Figure 3C vs. Table 2). Nonetheless, gene expression rates of both markers were already reduced compared to the unstimulated cells. 

#### 2.2.2. PBMCs and Osteoblasts

In order to mimic a more physiological situation, we established a co-culture of adherent PBMC cultures and osteoblasts. The purpose of these initial experiments was to analyse whether a co-culture between both cell types could reveal additional or different mechanisms and processes to those observed in the single cell cultures in response to CoCr29Mo6 ion treatment, i.e., whether the co-existence of both cell types per se had an impact on induction of the osteolytic processes. The co-culture was done in trans-well experiments where osteoblasts were seeded in inserts and adherent PBMCs were cultured in 12-well plates. Both cell types were cultivated in their respective cell culture medium whereas a mixing of both media can be assumed. The mixing had no influence on cell viability and differentiation which was analysed in preliminary experiments. In the co-culture, we exposed both cell types with 200 µg/L ion solution and investigated the differentiation and cytokine expression rates via qRT-PCR. These results were then compared to stimulated single cell cultures. 

**Figure 4:**
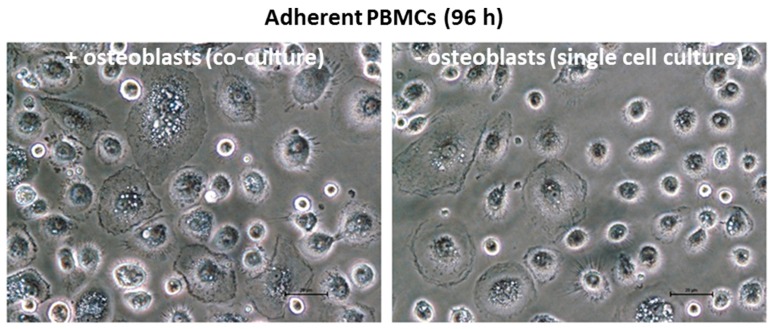
Light microscopy images of adherent PBMCs (scale bar: 20 µm).

The observation of the cell cultures using light microscopy suggested that treatment with the ion solution had an effect on the attachment of the monocytes and their differentiation to macrophages as an increase of sprawling cells was visible in the co-culture experiments (Figure 4). However, the number of these cells was not quantified. 

Differences between co-culture and single cultures in the response to metal ion exposure were also observed with regard to the gene expression analysis. In contrast to the single cell culture of human osteoblasts, we determined a significant decrease in *Col1A1* mRNA levels (*p* = 0.029) when osteoblasts were in contact with PBMCs (Table 3). Regarding *MMP1* gene expression, only a slight increase could be shown in osteoblasts whereas in PBMCs, a significant upregulation (*p* = 0.029) was detected (Table 3). Moreover, in both cell types, significantly increased *TIMP1* mRNA levels (both: *p* = 0.029) compared to the single cell culture were measured. *TIMP1* expression rates were even higher than those for *MMP1*. In the co-culture experiment, the mRNA levels of the cytokines *IL-6*, *IL-8*, and *TNF-α* were significantly increased (all: *p* = 0.029) in both cell types whereas *MCP-1* expression rates were significantly elevated in human osteoblasts only (*p* = 0.029). Interestingly, human osteoblasts in co-culture with PBMCs showed decreased *RANKL* but significantly increased *OPG* (*p* = 0.029) expression rates. The *RANK* gene expression of PBMCs was not affected in the co-culture system, whereas *TRAP5b* mRNA was highly induced (*p* = 0.029) (Table 3). 

## 3. Discussion

Adverse tissue effects could be mediated by degradation products from commonly used orthopaedic materials. In case of articulating surfaces, these degradation products are mainly the result of wear and corrosion, and comprise particles, inorganic salts or oxides, and free metal ions due to various forms of electrochemical corrosion [22]. To become bioactive and destructive, metal ions must react with biomolecules to induce osteolysis and aseptic loosening of endoprostheses [8]. In patients with high amounts of Co and Cr ions, an enhanced level of oxidative stress was seen which is known to be associated with tissue damage and necrosis [9,10,22]. In our experiments, we did not observe negative effects of metal ion exposure on cell metabolism and proliferation. These results were corroborated by the findings that cellular apoptosis was not influenced in both cell types. However, these investigations were performed in the adherent cells only. In contrast, necrosis was determined in the supernatant of cells and was significantly elevated in single cell cultures of human osteoblasts and PBMCs. In aseptic loosening of implants, the inflammation is mainly driven by activated macrophages which secrete several pro-osteolytic mediators such as *TNF-α*, *IL-1β*, *IL-6*, and *IL-8* [23,24]. This results further in monocyte recruitment, suppression of osteoblastic differentiation, and the induction of osteoclastogenesis. Activation of macrophages is therefore considered to be the key component in peri-implant osteolysis [23]. In order to study macrophage activation in vitro, we allowed adherence of monocytes from isolated, human PBMCs on cell culture plastic over a period of 72 h [25] before exposure of the adherent cells with metal ions. After a further 96 h of ion treatment, we detected sprawling cells suggesting a differentiation of monocytic cells to macrophages. This effect was more enhanced in co-cultures with human osteoblasts. In contrast, we did not observe these distinctive effects when the adherent PBMCs were treated directly (without prior adherence period) with metal ions in the presence of non-adherent PBMCs. The observation that direct exposure seemed to suppress the sprawl of the adherent cells, i.e., their differentiation, was mirrored by the downregulated mRNA levels of relevant cytokines in adherent/non-adherent PBMCs indicating opposing effects of metal ions in undifferentiated cells. These results were contrary to our expectations since it was reported that macrophages and lymphocytes are directly involved in wear-mediated inflammatory reactions [9]. Interestingly, immunosuppressive effects of Co-Cr particles [26,27] were already reported by Wang et al. who showed that these particles inhibited lymphocyte proliferation and the production of LPS-stimulated immunoglobulins [26]. A similar immunosuppressive effect might have been triggered by the ions in our experiments. In contrast, the significantly higher gene expression levels of inflammation markers *IL-6*, *IL-8*, *MCP-1*, and *TNF-α* in co-cultured osteoblasts and adherent PBMCs especially compared to the partly already elevated expression in the single cell cultures suggests that not only macrophages and lymphocytes are involved in wear-mediated inflammatory reactions [9], but that the complex interplay between all cell types drives the inflammation process. Indeed, the higher gene expression levels of cytokines in osteoblasts compared to PBMCs support the direct involvement of osteoblastic cells in inflammatory processes in response to corrosive end products. However, for future studies in co-cultured cells it may be important to investigate different doses of Co and Cr, since our previous studies in osteoblasts in single cultures indicated an inverse relationship between the metal ion dose and cytokine expression [28]. 

With regard to the activation of osteoclastogenesis as part of aseptic loosening, our results show that metal ions are likely to induce osteoclastogenesis in a *RANKL*-independent pathway. In the context of elevated cytokine expression in the PBMC-osteoblast co-culture. the report from Kudo et al., that proinflammatory cytokines such as *TNF-α* and *IL-6* are able to induce osteoclast formation, is of interest [29]. Based on induced gene expression levels of *TNF-α* and *IL-6* as well as *TRAP5b* in the co-culture, an induction of osteoclastogenesis in response to ion exposure can be assumed [22]. However, longer cultivation times should be considered for future experiments to allow for complete osteoclast differentiation. 

Besides the activation of osteoclastogenesis in peri-prosthetic tissue, the inflammatory mediators *TNF-α*, *IL-1β*, *IL-6*, and *IL-8* are able to inhibit osteoblast function [24]. Indeed, we observed a significant downregulation of *Col1A1* of osteoblasts co-cultured with PBMCs. This downregulation is in accordance with other studies indicating a reduction of osteoblastic differentiation by downregulated collagen type 1 synthesis rates as well as *ALP* activity in response to Co and Cr ions [30,31]. However, this is in contrast to our single cell experiments showing a significant upregulation for *Col1A1* with a subsequent increase of the protein after exposure with a mixture of 200 µg/L ion solution. These results indicate differing effects of metal ions on cellular behavior in single and co-cultured cell experiments, thus proving the importance of co-culture models in vitro. The different cell types can interact with each other by the release of several mediators in response to ion exposure. This interaction is realised mainly by the soluble forms of cytokines and chemokines. However, analysing the amounts of these soluble proteins proves a major challenge in co-cultures. Analysing the intracellular protein with Western Blot provides little information about the amounts that are released from the cells while the measurement of proteins in supernatants with ELISA techniques does not allow us to determine the cell type that released the protein. Therefore, protein measurements are recommended in single cell cultures. However, since we did not observe distinct differences between treated and untreated cells in the single cell experiments, we decided against measuring protein contents. Nevertheless, in further studies, the analysis of cytokine release on the protein levels should be taken into consideration. 

The direct analysis of proteins is also important when determining the impact of *MMP* expression on bone resorption processes. The significantly elevated *MMP1* expression levels in the osteoblast-PBMC co-cultures were counteracted by even higher *TIMP1* expression rates. While this gives an indication that bone resorption processes were not induced in the co-culture, only the determination of the *MMP1*/*TIMP1* ratio on activated protein levels can provide a definitive answer.

## 4. Conclusions

Our results indicate that the CoCr29Mo6 ion solution had only marginal effects on the expression of pro-osteolytic mediators in human osteoblasts and PBMCs alone, whereas the co-culture may provide a comprehensive model to study processes involved in osteolysis and bone loss in response to Co and Cr ions. In future studies, long-term experiments will be designed to analyse the effects of metallic ions on osteoclast formation and activation. Moreover, the importance of co-culture models in evaluating the systemic osteolytic response to wear products in vitro will be further tested. 

## 5. Materials and Methods

### 5.1. Isolation of Human Osteoblastic Cells and PBMCs

Isolation of human primary osteoblasts (2 female donors: mean age 76 years ± 3 years; 4 male donors: mean age 62 years ± 3 years) was performed under sterile conditions from the bone marrow of femoral heads according to the protocol of Lochner et al. [3] after the agreement of patients undergoing primary hip replacements (Local Ethical Committee AZ: 2010-10). Spongiosa material was extracted from the inside of femoral heads and washed three times in phosphate-buffered saline (PBS, Biochrom AG, Berlin, Germany). Afterwards enzymatic digestion with collagenase A and dispase II (both from Roche, Penzberg, Germany), the material was filtered through a cell strainer (70 µm pores, BD Bioscience, Bedford, UK). The cell suspension was centrifuged at 118× *g* for 10 min and the cell pellet was resuspended in cell culture medium. Cells were cultivated in a special formulation of Dulbecco’s modified Eagle medium (DMEM, Biochrom AG) containing 10% fetal calf serum (FCS, Gibco^®^ Invitrogen, Paisly, UK), 1% penicillin/streptomycin, 1% amphotericin b, 1% hepes buffer, and the osteogenic additives l-ascorbate-2-phosphate (50 µg/mL), β-glycerophosphate (10 mM), as well as dexamethasone (100 nM) (all: Sigma-Aldrich, Munich, Germany) at 37 °C and 5% CO_2_. The osteoblastic phenotype was analysed by alkaline phosphatase staining (with fuchsin +substrate-chromogen; DAKO, Hamburg, Germany). Cells from passage 3 were harvested for the cell culture experiment (see Section 5.6, Experimental design). Briefly, cells were washed with PBS, trypsinised, and centrifuged at 118× *g*. Afterwards, a cell number of 10,000 cells (in duplicates) was transferred into a well of a 24-well cell culture plate allowing cell adherence over 24 h at 37 °C and 5% CO_2_.

Human PBMCs were isolated from buffy coats of healthy donors (Local Ethical Committee AZ: A2011-140) which were provided by the Institute of Transfusion Medicine, University Medical Center Rostock anonymously. Isolation of PBMCs was done by using density gradient centrifugation at 320× *g* and 230× *g* with Histopaque^®^-1077 (Sigma Aldrich, Munich, Germany) according to the manufacturer’s instructions. The interphase, containing a mixture of lymphocytes and monocytes, was extracted by means of a Pasteur pipette. After washing the cell suspension two times with PBS, cells were cultivated in Cell-Repellent culture plates (Greiner bio-one, Frickenhausen, Germany) with 1 × 10^7^ cells per well using Roswell Park Memorial Institute (RPMI) 1640 medium (Biochrom AG) supplemented with 5% FCS (Gibco^®^ Invitrogen), 1% penicillin/streptomycin, and 1% l-glutamine (both: Sigma-Aldrich) at 37 °C and 5% CO_2_. After seven days in Cell-Repellent culture plates, the cell suspension was centrifuged at 118× g and a cell number of 4 × 10^5^ (in duplicates) was transferred into a well of a standard 24-well cell culture plate (if not otherwise stated) allowing cell adherence over 72 h at 37 °C and 5% CO_2_.

### 5.2. Cell Viability

The viability of human osteoblasts and PBMCs after exposure to metal ions was determined by the metabolic activity assay WST-1 (Roche, Penzberg, Germany), DNA quantification, and live/dead stainings.

For the WST-1 test, the ion solution was removed and cells were incubated with a defined volume of WST-1/medium reagent (ratio 1:10) at 37 °C and 5% CO_2_ for 30 min. Afterwards, supernatants of the respective culture condition were transferred into 96-well cell culture plates (in duplicates) to determine the absorption at 450 nm (reference wave length: 630 nm) in a microplate reader (Dynex Technologies, Denkendorf, Germany).

DNA isolation of treated and untreated osteoblasts and adherent PBMCs was done by the peqGOLD Tissue DNA Mini Kit (Peqlab, Erlangen, Germany), according to the manufacturer’s instructions. Therefore, samples were lysed under denaturing conditions and applied on PerfectBind DNA columns which reversibly bind DNA. After washing away cellular debris and other proteins, the DNA was eluted in Aqua dest. Finally, DNA concentrations were measured with the Qubit Fluorometer according to the manufacturer’s recommendations (Invitrogen, Darmstadt, Germany).

Live-dead staining (Invitrogen) was carried out to distinguish vital and dead cells. The fluorescence dye calcein AM is membrane-impermeable, resulting in a bright green fluorescence (ex/em: 495/515 nm) of cells. In contrast, ethidium homodimer 1 enters damaged cells and binds at DNA resulting in a red fluorescence (ex/em: 495/635). Both dyes were dissolved in PBS and cells were incubated at room temperature in the dark. Cells were examined under a fluorescence microscope (Nikon ECLIPSE TS100, Nikon GmbH, Duesseldorf, Germany) at the same position for live and dead cells. Afterwards, an overlay of both pictures was done using Adobe Photoshop CS6 (Adobe Systems Software Ireland Ltd., Dublin, Ireland). 

### 5.3. Determination of Apoptosis and Necrosis

The enrichment of mono- and oligonucleosomes within osteoblastic and PBMC cell cultures was quantified after exposure with metal ions in order to determine the apoptosis and necrosis rate of human osteoblasts and PBMCs. Therefore, the Cell Death Detection ELISA^PLUS^ Kit (Roche) was used following the instructions of the manufacturer. To determine apoptosis, cells were lysed for 30 min at room temperature. To determine necrosis, the supernatants of cells were used. The absorbance was measured at 405 nm (reference wave length: 490 nm) using a microplate reader (Dynex Technologies). In accordance to the manufacturer’s instructions, the apoptosis and necrosis rate was then normalised to the untreated control at each time point.

### 5.4. Gene Expression Analyses

For gene expression analyses, the RNA of treated and untreated cells was isolated with the Direct-zol Kit (Zymo Research, Freiburg, Germany) according to the supplier’s recommendation. For cDNA synthesis, 10 to 50 ng RNA was reversed transcribed using the High Capacity cDNA Kit (Applied Biosystems, Forster City, CA, USA) according to the manufacturer’s instructions. 

Relative quantification of target cDNA levels was done by quantitative realtime PCR (qTower 2.0, Analytik Jena, Jena, Germany) using the innuMix qPCR MasterMix SyGreen (Analytik Jena) and the primers (Sigma-Aldrich) described in Table 4. cDNA sequences were generated using the NCBI database. PCR analysis was performed under the following conditions: 2 min at 95 °C, 40 cycles of 95 °C (5 s) and 65 °C (25 s). The reactions were performed in duplicates. The relative expression of each mRNA compared with the housekeeper gene HPRT was calculated by the equation ∆Ct = Ct_target_ − Ct_HPRT_. The relative amount of target mRNA in the unstimulated cells and treated cells was expressed as 2^(−∆∆Ct)^, where ∆∆Ct_treatment_ = Ct_target_ − Ct_control_.

### 5.5. Pro-Collagen Type 1 Synthesis Rate

The synthesis rate of pro-collagen type 1 by human osteoblasts was determined using the C1CP ELISA (Quidel, Marburg, Germany). For this instance, the supernatants of treated and untreated osteoblasts were collected and stored at −20 °C. The ELISA was performed to the manufacturer’s recommendations. Absorbance was measured at 405 nm (reference wave length: 630 nm) using a microplate reader (Dynex Technologies). The respective protein content was calculated using a standard curve. Afterwards, the C1CP protein contents within the probes were normalised to the overall protein content which was quantified using the Qubit Protein Assay Kit and Qubit 1.0 (both: Invitrogen) according to the manufacturer’s instructions.

### 5.6. Experimental Design

For the cell culture experiments, an ion solution from a CoCr29Mo6 alloy was prepared. Solid material samples served as an anode against hydrogen bridge electrodes in a corrosion measuring cell. The released metal ions were dissolved in PBS and the content of all ions in the stock solution was analysed by a high resolution, magnetic sector field Inductively Coupled Plasma-Mass Spectrometry (ICP-MS) instrument (Element-2, Thermo Scientific, Bremen, Germany) before and after the in vitro experiments. The contents are shown in Table 5. The ion solution was heat-treated to eliminate endotoxins which was proven by the Limulus Amebocyte Lysate (LAL) test (Lonza, Cologne, Germany).

For each stimulation experiment, a mixture of 200 µg/L ion solution was prepared in the respective cell culture medium (with additives). Cells were exposed to the ion solution over 48 h and 96 h. Unstimulated cells served as controls. We determined the influence of metal ions on cell viability and apoptosis/necrosis in human osteoblasts and PBMCs. Moreover, gene expression rates of osteoblastic differentiation (*Col1A1*, *ALP*, *OC*), bone remodelling (*RANKL*, *OPG*) markers, and osteoclastic differentiation markers (*RANK*, *TRAP5b*), as well as different cytokines and chemokines (*IL-6*, *IL-8*, *MCP-1*, *TNF-α*, *MMP1*, *TIMP1*) in single cultures were analysed. Additionally, the influence of ions on monocytic differentiation from adherent cells was analysed in PBMCs. For this purpose, we transferred 4 × 10^5^ isolated PBMCs per well (in duplicates) in RPMI1640 with additives in a standard 24-well culture plate and exposed cells with a 200 µg/L ion solution. Unstimulated cells served as controls. After 48 h and 96 h, we determined the metabolic cell activity and the gene expression rates of different cytokines (*IL-6*, *IL-8*, *MCP-1*, *TNF-α*) in adherent and non-adherent cells. Osteoclastic differentiation markers (*RANK*, *TRAP5b*) were also studied in adherent PBMC cultures. For analysing cell viability and gene expression of non-adherent PBMCs, we transferred the cells in the supernatants in 1.5 mL tubes and centrifuged them at 118× *g*. The remaining cell pellet was resuspended in WST-1 reagent to determine the metabolic activity. After 30 min, the cell-reagent suspension was centrifuged and the supernatants were transferred as duplicates in 96-Well plates to determine the absorbance as previously mentioned in Section 5.2. The remaining cell pellets were washed two times in PBS. Afterwards, RNA was isolated for further gene expression analyses (see Section 5.4).

In another experimental set-up, we prepared a co-culture between human osteoblasts and PBMCs. We seeded monocytes (*n* = 4; 4 × 10^5^ cells per well in RPMI1640 with additives) in 24-well cell culture plates and allowed adherence over 72 h. Human osteoblasts (*n* = 1, 10,000 cells/well in DMEM with additives) were seeded into cell culture inserts (Thincert, Greiner bio-one). After 24 h of adherence, the cell-seeded inserts were transferred into the cell culture plate with the PBMCs. Both cell types were exposed to metal ions (200 µg/L) over 96 h. Simultaneously, ion exposed single cell cultures of adherent PBMCs in the cell culture plate and osteoblasts in the inserts were also maintained for 96 h and served as controls. In this experimental set-up, we determined the influence on *Col1A1* expression rates in osteoblasts as well as cytokine expression rates and bone remodelling markers in both cell types.

### 5.7. Data Illustration and Statistical Analysis

If not otherwise stated, data are presented as box plots. Boxes denote interquartile ranges, horizontal lines within the boxes denote medians, and whiskers denote minimum and maximum values. For all analyses, the cultures of human osteoblasts and monocytes from a minimum of four independent donors were used (in duplicates). Since the data obtained were not normally distributed, the statistical significance between two datasets was calculated with the Mann-Whitney-U test using SPSS 20 (IBM Deutschland, Ehningen, Germany). The level of significance was set to a *p*-value less than or equal to 0.05 (*p* ≤ 0.05).

## Figures and Tables

**Table 1:** Expression of differentiation markers in human osteoblasts and adherent PBMCs after exposure with 200 µg/L CoCr29Mo6 ion solution. Cells were transferred into standard culture plates allowing adherence over 24 h (osteoblasts) or 72 h (PBMCs). Afterwards, the cell culture medium was replaced by the ion solution. After 48 h and 96 h, RNA was isolated and reverse transcribed. For protein measurements (C1CP), supernatants of osteoblasts were used. Gene expression analysis was done by quantitative Realtime PCR (qRT-PCR). Data are shown as median and minimum/maximum values (*n* ≥ 4; all in %). Significances between groups were calculated with the Mann-Whitney-U-Test (* *p* ≤ 0.05, ** *p* < 0.01, treated vs. untreated; **^#^** *p* ≤ 0.05, 48 h vs. 96 h).

Differentiation Marker	Osteoblasts		Adherent PBMCs	
	48 h	96 h	48 h	96 h
***Col1A1***	***95*** ***(57; 119)***	214 ** (104; 411)	*****-*****	*****-*****
**C1CP (protein)**	**80** (66; 102)	**136** (85; 179)	**-**	**-**
***ALP***	**91** (42; 124)	**116** (76; 141)	**-**	**-**
***OC***	**110** (82; 248)	**90** (72; 193)	**-**	**-**
***OPG***	**95** (36; 162)	**107** (19; 139)	**-**	**-**
***RANKL***	**286** (5; 2026)	**46** (14; 837)	**-**	**-**
***RANK***	**-**	**-**	**56** *^,#^ (50; 76)	**36** *^,#^ (35; 49)
***TRAP5b***	**-**	**-**	**5** * (2; 9)	**7** * (2; 11)

**Table 2:** Gene expression analyses of pro-osteolytic mediators in human osteoblasts and PBMCs after exposure with 200 µg/L CoCr29Mo6 ion solution. Cells were transferred into standard cell culture plates allowing adherence over 24 h (osteoblasts) or 72 h (PBMCs). Afterwards, the cell culture medium was replaced by the ion solution. After 48 h and 96 h, RNA was isolated and reverse transcribed. Gene expression analyses were done by qRT-PCR. Data are shown as median and minimum/maximum values (*n* = 4; all in %). Significances between groups were calculated with the Mann-Whitney-U-Test (* *p* ≤ 0.05, ** *p* < 0.01, treated vs. untreated).

Pro-Osteolytic Mediators	Osteoblasts		Adherent PBMCs	
	48 h	96 h	48 h	96 h
***MMP1***	**95** (36; 162)	**52** (36; 144)	**234** (92; 338)	**161** (23; 778)
***TIMP1***	**140** * (110; 299)	**110** (66; 269)	**91** (30; 337)	**122** (89; 675)
***IL-6***	**276** ** (117; 546)	**99** (75; 204)	**120** (14; 659)	**94** (56; 166)
***IL-8***	**143** (48; 247)	**58** * (50; 73)	**404** * (127; 1594)	**246** (34; 4104)
***TNF-α***	**104** (51; 165)	**198** (51; 435)	**129** (96; 312)	**83** (76; 130)
***MCP-1***	**120** (42; 149)	**101** (68; 130)	**29** * (19; 41)	**703** (13; 1628)

**Table 3:** Evaluation of the effects of CoCr29Mo6 ions on co-cultures of PBMCs and osteoblasts in comparison to single-cultured cells (PBMCs: *n* = 4; PBMCs; osteoblasts: *n* = 1 (same osteoblastic donor for all PBMC cultures)). PBMCs were transferred into standard culture plates allowing adherence over 72 h. Osteoblasts were seeded into cell culture inserts. After 24 h, cell culture inserts were transferred into the PBMC cultures and the respective cell culture medium was replaced by the ion solution. After 48 h and 96 h, RNA was isolated and reverse transcribed. Gene expression analyses of the differentiation markers and cytokines in the osteoblasts and PBMCs were done by qRT-PCR. Data are shown as median and minimum/maximum values (all in %). Significances between groups were calculated with the Mann-Whitney-U-Test (* *p* ≤ 0.05, treated vs. untreated).

Differentiaton or Pro-Osteolytic Mediators	Osteoblasts	Adherent PBMCs
***Col1A1***	**31** * (24; 49)	****-****
***RANKL***	**2** (1; 5)	****-****
***OPG***	**155** * (118; 206)	****-****
***RANK***	****-****	**85** (47; 108)
***TRAP5b***	****-****	**10,649** * (9263; 12,099)
***MMP1***	**128** (111; 307)	**285** * (212; 361)
***TIMP1***	**2950** * (307; 8706)	**2573** * (978; 3490)
***IL-6***	**5,306,604** * (3,684,037; 5,701,287)	**1093** * (700; 1315)
***IL-8***	**4,089,451** * (1,769,434; 5,611,116)	**31,637** * (21,602; 46,144)
***TNF-α***	**489,124** * (194,021; 741,895)	**603** * (258; 887)
***MCP-1***	**207,580** * (169,374; 267,626)	**654** (98; 1277)

**Table 4:** cDNA target sequences for qRT PCR.

Primer	Forward (Sequence 5′-3′)	Reverse (Sequence 5′-3′)
Alkaline phosphatase (*ALP*)	cattgtgaccaccacgagag	ccatgatcacgtcaatgtcc
Collagen 1 (*Col1A1*)	acgaagacatcccaccaatc	agatcacgtcatcgcacaac
Hypoxanthine-guanine phosphoribosyltransferase (HPRT)	ccctggcgtcgtgattagtg	tcgagcaagacgttcagtcc
Interleukin 6 (*IL-6*)	tggattcaatgaggagacttgcc	ctggcatttgtggttgggtc
Interleukin 8 (*IL-8*)	tctgtgtgaaggtgcagttttg	atttctgtgttggcgcagtg
Matrix metalloproteinase 1 (*MMP1*)	agagcagatgtggacatgc	tcccgatgatctcccctgac
Monocyte chemotactic protein 1 (*MCP-1*)	ccgagaggctgagactaacc	ggcattgattgcatctggctg
Osteocalcin (*OC*)	ggtgcagcctttgtgtcc	tcagccaactcgtcacagtc
Osteoprotegerin (*OPG*)	tggattcaatgaggagacttgcc	ctggcatttgtggttgggtc
Receptor activator of nuclear κ-b (*RANK*)	agaaaaccaccaaatgaacccc	gccacaagcctcattgatcc
Receptor activator of nuclear κ-b-Ligand (*RANKL*)	agaagccaccaaagaattgcag	accatcgctttctctgctctg
Tartrate-resistant acid phosphatase 5b (*TRAP5b*)	gggagatctgtgagccagtg	gtccacatgtccatccaggg
Tissue inhibitor of metallo-proteinase 1 (*TIMP1*)	attgctggaaaactgcaggatg	gtccacaagcaatgagtgcc
Tumour necrosis factor α (*TNF-α*)	gttgtagcaaaccctcaagctg	gaggtacaggccctctgatg

**Table 5:** Total ion concentrations of the CoCr29Mo6 stock solution as well as in the experimental solution (200 µg/L in total).

Content in	Co	Cr	Mo	Ni
stock solution	12.0 ± 2.4 mg/L	3.9 ± 0.6 mg/L	0.9 ± 0.1 mg/L	1.3 ± 0.6 mg/L
experimental solution	120 ± 24 µg/L	39 ± 5.7 µg/L	8.8 ± 1.1 µg/L	12.8 ± 6.0 µg/L
